# 22q11.21 Deletions: A Review on the Interval Mediated by Low-Copy Repeats C and D

**DOI:** 10.3390/genes16010072

**Published:** 2025-01-09

**Authors:** Veronica Bertini, Francesca Cambi, Annalisa Legitimo, Giorgio Costagliola, Rita Consolini, Angelo Valetto

**Affiliations:** 1 Section of Cytogenetics, Oncology Department, Azienda Ospedaliero-Universitaria Pisana, 56126 Pisa, Italy; f.cambi@ao-pisa.toscana.it (F.C.); a.valetto@ao-pisa.toscana.it (A.V.); 2 Section of Clinical and Laboratory Immunology, Pediatric Unit, Department of Clinical and Experimental Medicine, University of Pisa, 56126 Pisa, Italyrita.consolini@med.unipi.it (R.C.); 3 Section of Pediatric Hematology and Oncology, Azienda Ospedaliero-Universitaria Pisana, 56126 Pisa, Italy; giorgio.costagliola@hotmail.com

**Keywords:** LCR22s, 22q11.2DS, nested deletion, central deletion, renal urinal anomalies, cardiac defects, neurological disorders, *CRKL*, CNV

## Abstract

22q11.2 is a region prone to chromosomal rearrangements due to the presence of eight large blocks of low-copy repeats (LCR22s). The 3 Mb 22q11.2 “typical deletion”, between LCR22-A and D, causes a fairly well-known clinical picture, while the effects of smaller CNVs harbored in this interval are still to be fully elucidated. Nested deletions, flanked by LCR22B-D, LCR22B-C, or LCR22C-D, are very rare and are collectively described as “central deletions”. The LCR22C-D deletion (CDdel) has never been separately analyzed. In this paper, we focused only on CDdel, evaluating its gene content and reviewing the literature and public databases in order to obtain new insights for the classification of this CNV. At first glance, CDdels are associated with a broad phenotypic spectrum, ranging from clinically normal to quite severe phenotypes. However, the frequency of specific clinical traits highlights that renal/urinary tract abnormalities, cardiac defects, and neurological/behavioral disorders are much more common in CDdel than in the general population. This frequency is too high to be fortuitous, indicating that CDdel is a predisposing factor for these phenotypic traits. Among the genes present in this interval, *CRKL* is an excellent candidate for cardiac and renal defects. Even if further data are necessary to confirm the role of CDdels, according to our review, this CNV fits into the class of ‘likely pathogenic’ CNVs.

## 1. Introduction

22q11.2 is a region prone to chromosomal rearrangements (i.e., deletions and duplications) due to the presence of eight large blocks of low-copy repeats (LCR22s: LCR22-A to LCR22-H). In this paper, we focused on 22q11.2 deletions flanked by LCR22-C and D, since their clinical meaning has not yet been fully clarified.

The interpretation of the effects of a copy number variant (CNV), such as the 22q11.2C-D deletion, is a multi-step process that requires mainly the evaluation of its genomic content and the analysis of the similar imbalances in the medical literature. This process leads to a five-tier classification (pathogenic, likely pathogenic, uncertain significance, likely benign, and benign).

The study of 22q11.2C-D deletion was performed according to the recent semi-quantitative point-based scoring system developed by the American College of Medical Genetics and Genomics (ACMG) and the Clinical Genome Resource (ClinGen) [1].

## 2. Architecture of the 22q11.2 Region

**Figure 1:**
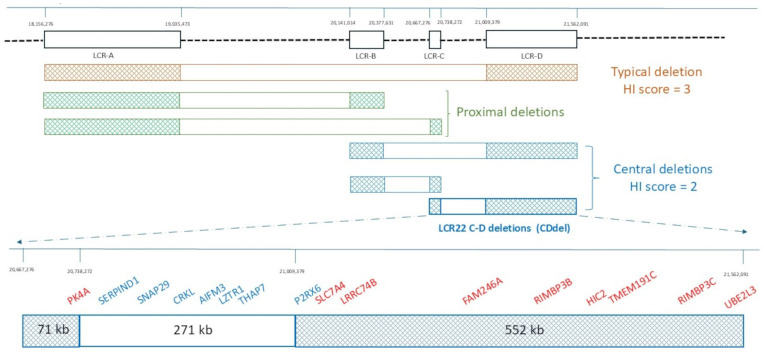
Schematic representation of typical, proximal, and central 22q11.2 deletions. Low-copy repeats (LCRs) from A to D are depicted as dark squares. Their localization refers to GRCh38/hg38. The orange bar represents the typical 22q11.2 deletion, the green ones the proximal deletions. and the blue ones the central deletions. For each group of deletions, the genomic haploinsufficiency (HI) score is given (https://www.ncbi.nlm.nih.gov/projects/dbvar/clingen/) (accessed on 28 November 2024). At the bottom, a detailed representation of the LCR22C-D deletion (CDdel) and its gene content is shown.

The 22q11.2 chromosome region contains eight large blocks of low-copy repeats (LCR22s: LCR22-A to LCR22-H) that are predisposed to meiotic non-allelic homologous recombination and lead to genomic imbalances (Figure 1) [2,3].

Deletions involving the four proximal LCRs (from LCR22A to LCR22D) cause the “22q11.2 deletion syndrome” (22q11.2DS), a heterogeneous multisystemic condition related to an impaired development of the third and fourth pharyngeal arches [2,3,4].

The vast majority of 22q11.2DS cases have a ~3 Mb deletion, known as the “**typical or proximal typical deletion**”, flanked by LCR22A-D (Figure 1). Individuals with atypical deletions due to a different combination of LCR22s or with breakpoints not falling into LCRs have also been reported [3].

About 5–10% of the cases show “**proximal nested deletions**” flanked by LCR22A-B or LCR22A-C, whose phenotype is indistinguishable from the “proximal typical deletion” (Figure 1). The clinical meaning of these intervals (LCR22A-D, LCR22A-B, and LCR22A-C) is also well-documented by ClinGen (https://www.ncbi.nlm.nih.gov/projects/dbvar/clingen/) (accessed on 28 November 2024), a publicly available resource that collects evidence supporting and/or refuting the “dosage sensitivity” (DS) for genomic regions and provides a “haploinsufficiency” (HI) DS score. For these intervals, the HI DS score is 3, which corresponds to “sufficient evidence for dosage pathogenicity”.

Nested deletions, flanked by LCR22B-D, LCR22B-C, or LCR22C-D, are very rare and collectively described as “**central deletions**” with a HI DS score of 2, corresponding to “emerging evidence for haploinsufficiency” (https://www.ncbi.nlm.nih.gov/projects/dbvar/clingen/) (accessed on 28 November 2024) (Figure 1).

The LCR22C-D deletion (hereinafter called “CDdel”) has never been separately analyzed. In this paper, we focused only on CDdel, evaluating its gene content and reviewing the literature and public databases in order to obtain new insights for the classification of this CNV.

## 3. Gene Content

A crucial step for classifying a CNV is to verify if it overlaps with any established or predicted HI score gene, and, in particular, with any gene associated with dominantly inherited disorders (https://www.ncbi.nlm.nih.gov/projects/dbvar/clingen/) (accessed on 28 November 2024) [1].

If we exclude the LCR22C and D flanking sequences, the LCR22C-D deletion spans approximately 271 kb (GRCh38/hg38, chr22: 20,738,272–21,009,379) and harbors seven protein-coding genes (Figure 1). Identifying the exact number of genes harbored in the LCR22C-D deletions may be a challenge, since it is difficult to exactly locate the breakpoints into the LCRC and D, due to the oligo/SNP low coverage of these regions in the array platforms. In view of this, the genes harbored in LCR22C (71 kb) and LCR22D (552 kb) were also evaluated, since it cannot be excluded that their deletion, if present, may affect the phenotype [2] (track “NCBI RefSeq genes” of UCSC https://genome.ucsc.edu/cgi-bin/hgGateway) (accessed on 28 November 2024) (Table 1).

Only two genes, *LZTR* (leucine zipper-like transcription regulator 1) and *SERPIND*1 (heparin cofactor II), are associated with dominantly inherited disorders and for none of them has the HI score been recorded (https://www.ncbi.nlm.nih.gov/projects/dbvar/clingen/) (accessed on 28 November 2024). Regarding *LZTR*, its pathogenicity results from mutational mechanisms different from the dosage sensitivity: “Noonan syndrome 10” is caused by dominant negative mutations, and “Schwannomatosis-2” is the result of a multi-step pathway involving several others genes [5,6]. Nothing is known about the mechanism underlying the pathogenicity of *SERPIND1*.

None of the few available HI of CDdel genes reaches a score of 3 (sufficient evidence for dosage pathogenicity) (Table 1).

Even if it reaches a HI score of only 1 (little evidence of pathogenicity), *CRLK* (CRK like proto-oncogene, adaptor protein) is the most interesting gene, according to studies in mice and humans (https://www.ncbi.nlm.nih.gov/projects/dbvar/clingen/) (accessed on 28 November 2024) (Table 1).

Knockout mice with *Crkl* haploinsufficiency shows some clinical features observed in the 22q11.2 DS, such as cardiovascular and genitourinary defects [7,8,9]. Moreover, in a vast cohort of individuals with 22q11.2DS, common variants on the not-deleted allele are associated with a moderate increased risk for conotruncal heart defects (CTDs). These variants are located in a 350 kb region, largely within the LCR22C-D interval; the top associated variants lie in putative regulatory regions of *CRKL*, strengthening the involvement of this gene in cardiac pathologies [10]. A role of *CRKL* in renal anomalies has been suggested by variants in domains evolutionarily conserved, found in five individuals with renal agenesis or hypodysplasia [9].

Since there are no genes with a significant HI score in the region, we evaluated two other HI predictors, the gnomAD probability of loss-of-function intolerance score (pLI) and the DECIPHER HI index (%HI) [1]. Due to the intrinsic limitations of these tools, only genes with a pLI score ≥ 0.9 and %HI index of ≤10% can be considered to have supportive evidence of HI. As shown in Table 1, none of the genes in the LCR22C-D interval fall into this category.

Taking into account all these data (the HI score evaluation, HI predictors, and mechanisms of pathogenicity of genes associated with dominantly inherited disorders), neither the pathogenicity nor benignity of CDdel can be established.

## 4. Long-Distance Effects of CNVs

As known, CNVs may have long-distance effects through chromatin structure alterations or epigenetic modifications [11,12].

Topologically associated domains (TADs) can be defined as linear units of chromatin that fold, as discrete three-dimensional (3D) structures tending to favor internal chromatin interactions [11]. TADs constitute functional units in the genome, important for the correct regulation of gene expression, since they co-localize regulatory elements with their target genes. TAD boundaries are structural barriers necessary to ensure this 3D genomic organization and their disruption is found to be associated with a wide range of diseases [11]. The LCRC-D interval mostly overlaps with a 350 kb region (GRCh38/hg38, chr22: 20,607,741–20,958,141) within a single TAD, as determined by an analysis of the chromatin structure by high-throughput chromatin conformation capture (Hi-C), (track “HapMap” of UCSC, https://genome.ucsc.edu/cgi-bin/hgGateway) (accessed on 28 November 2024). According to these data, CDdel disrupts a TAD boundary, but whether this has any long-range effect on gene expression has not yet been studied.

Recently, a DNA methylation episignature in the 22q11.2DS has been identified [12]. Both the typical and proximal 22q11.2 deletions show a unique and highly specific episignature, that may impact the expression of genes outside the 22q11.2 region, potentially relevant in the pathogenesis of the 22q11.2DS phenotype; however, this altered methylation pattern is not present in either the LCR22B-D or LCR22C-D deletions, suggesting that central deletions are entities distinct from 22q11.2DS, at least on the basis of the episignature.

## 5. CDdel Cases from Published Literature and Public Databases

A thorough review of public databases and medical literature was performed to identify cases with CDdel and to evaluate clinical features reported along with their relative frequencies.

Cases were excluded from our analysis if they showed (1) deletions overlapping CDdel, but with at least one breakpoint beyond LCR-C or LCR-D; (2) additional CNVs or gene variants in other genome regions; or (3) no clinical phenotype description. Cohorts of cases with “central deletions” in which CDdel patients were indistinguishable from those with different intervals of deletions were not included (Figure 1) [13,14,15,16,17,18,19,20].

Taking into consideration these criteria, a cohort of 65 individuals with CDdel, including 56 symptomatic and 9 asymptomatic cases, were selected. Only the individuals with pathological features were reported in the Table 2 and identified with a progressive “P number” [8,9,19,21,22,23,24,25,26,27,28,29] (Decipher: https://www.deciphergenomics.org/) (accessed on 28 November 2024).

All their clinical features are described in Supplementary Tables S1 and S2, as well as the pathological signs investigated but not found (Supplementary Table S3). CDdels are associated with a broad phenotypic spectrum, ranging from a mild to a quite severe phenotype. It is likely that cases with significant and profound disorders are a consequence of additional genetic variants, since neither Whole-Exon Sequencing (WES) or Whole-Genome Sequencing (WGS) were performed in any of these patients.

The nine asymptomatic individuals, including four parents (parents of P24, P25, P43, and P46) and five cases (nsv588305, esv3893441, esv2678175, and nsv4277899) reported in DGV (https://dgv.tcag.ca/) (accessed on 28 November 2024) suggest that the frequency of this alteration is probably underestimated (Table 2).

As reported in Table 3, the pathological traits have been grouped in macro-areas and their frequency is reported as the ratio between the number of affected cases and the total number of selected cases. A detailed description of each clinical sign was also reported along with the number of individuals where the clinical trait has actually been evaluated and excluded.

A rigorous statistical analysis of the frequency of clinical features is precluded due to the small number of individuals, the variable accuracy of the phenotypic description, and the presence of familiar cases where the genetic background may infer the phenotypic outcome.

Nevertheless, this careful analysis of the CDdel phenotypes allowed us to identify clinical traits present with a higher or lower frequency.

### 5.1. More Frequent Pathological Traits

The most frequent pathological traits include (1) renal/urinary tract abnormalities, (2) cardiac defects, and (3) neurological/behavioral disorders.

The total number of CDdel cases presenting with these three phenotypic categories is higher than 65, since each of these aspects has been evaluated singularly in studies (or databases) where no other clinical characteristics were examined [9,24].

These pathological aspects have a different ‘weight’ on CNV classification: congenital cardiac or renal/urinary tract defects are considered “highly specific and well-defined” features, because they represent distinct phenotypic traits, often with a genetic etiology and a limited genetic heterogeneity; on the other hand, neurological/behavioral aspects are “non-specific” since they are more common in the general population, have more considerable genetic heterogeneity, and often have a non-genetic etiology. For these reasons, their contribution in defining the pathogenicity of a CNV is lower [1].

#### 5.1.1. Renal/Urinary Tract Abnormalities

Renal/urinary tract defects represent the most frequently evaluated clinical feature in CDdel cases. Some of these cases (P7, P8, P9, P28, P29, P30, and P34) were recruited through the search for structural variants in large cohorts affected by congenital anomalies of the kidney and urinary tract [9,24]. Querying the Children’s Hospital of Philadelphia’s “22q and You” database (https://www.chop.edu/centers-programs/22q-and-you-center/news) (accessed on 28 November 2024), two additional individuals (P35, P36) were found [9]. These works of research also led to the identification of nine CDdel people without renal and urinary tract abnormalities, including eight cases collected in the “22q and You” database and one person recovered from the population used as a control by Lopez-Riveira et al., 2017 [9]. Since the clinical features of these nine individuals were not known except for the absence of the renal–urinary phenotype, they were included in only this phenotypic category.

To summarize, as far as the renal/urinary anomaly group is concerned, a total of 74 people were considered (65 + 9). In 23/74 cases, they were present (31.1%), and they were excluded in 21.

The renal/urinary anomalies reported are quite heterogeneous; notably, most of the cases (15/23) show a quite severe phenotype, including renal agenesis (N = 9) or hypodysplasia (N = 6).

As mentioned above, according to studies in humans and mice, the CRKL gene could be responsible for the urinary tract defects [7,9]. The rather high frequency of these abnormalities, probably due to CRKL, makes CDdel a risk factor for renal/urinary tract defects.

#### 5.1.2. Cardiac Defects

As in 22.11.2DS, conotruncal defects (CTDs) are the most frequently reported alterations in CDdel cases (Table 3), when a cardiological evaluation is performed. T-box transcription factor 1 (TBX1) was identified as the main gene responsible for cardiac abnormalities in 22q11.2DS. However, this gene is not included in the CDdel region; thus, there should be other genes responsible for these defects. A good candidate is CKRL, since the Ckrl−/− knockout mice showed an altered heart development [7,8].

To summarize, cardiac defects were reported in 10/69 cases (14.5%) and excluded in 21. This frequency is lower than in 22q11.2DS syndrome (25–35%) [30], but higher than in the general population (approximately from 0.4 to 5% in live births) [31], highlighting that CDdels may also have a role as a predisposing factor for cardiac anomalies.

#### 5.1.3. Neurological/Behavioral Disorders

Among the selected 65 cases, the neurological/behavioral disorders were reported in 31 individuals. However, 6 additional cases (nssv3466483, nssv3482339, nssv3472863, nssv3482015, nssv13648688, and nssv580067) were added after a search in public databases that collect CNVs found in patients with Developmental Delay (DD) and/or Intellectual Disability (ID), such as the Copy Number Morbidity map and Clinical Genome Resource CNVs (track “Development Delay” and “ClinGen CNVs” of UCSC, https://genome.ucsc.edu/cgi-bin/hgGateway) (accessed on 28 November 2024).

This group collects a wide spectrum of clinical signs, ranging from mild to severe phenotypes. As stated above, these disorders are usually considered to have less “weight” in defining the pathogenicity of a CNV. Nevertheless, it can be inferred that CDdels represent a predisposing factor for the manifestation of neurological/behavior disorders, since they have been described in about half of the cases (37/71, 52.1%) (Table 3). In order to better analyze our data, this group was split into six macro-areas: (1) Developmental Delay (DD) and/or Intellectual Disability (ID), found in 27/71 (38%); (2) autism spectrum disorder (ASD) in 3/71 (4.2%); (3) attention-deficit/hyperactivity disorder (ADHD) spectrum disorder in 6/71 (8.5%); (4) behavioral and mood disorders in 5/71 (7%); (5) movement disorders in 7/71(9.9%); and (6) “heterogeneous” neurological signs in 12/71 (16.9%) (Table 3).

It is interesting to note that, in our cohort, different Neuro-Developmental Disorders (NDDs), including ID, DD, ASD, and ADHD, may concur in the same patient. This comorbidity is almost a constant feature of NDDs, with different NDDs probably being related to the variable expressivity of the same genetic alteration [32].

A search for candidate genes within the interval LCR22B-D highlighted that PI4KA (phosphatidylinositol 4-kinase alpha) is highly expressed in fetal and adult brain tissues, with particularly high levels in adult cerebral cortical tissues [33], but further data are necessary to confirm its role.

### 5.2. Less Frequent Pathological Traits

This section includes a miscellany of traits that are present in more than one person without reaching a significant frequency.

Most CDdel individuals show facial dysmorphisms, but their heterogeneity does not allow us to identify a shared gestalt as in the 22q11.2DS ones (Table S2). Palatal defects were reported only in six people (P2, P3, P18, P19, P22, and P27).

Various genital defects are showed in 7/65 cases (10.8%), with the cryptorchidism as the most prevalent anomaly (P14, P16, P28, and P33).

Skeletal abnormalities, observed in 13 individuals, were too heterogeneous to be considered a distinctive clinical trait.

Frequent infections, particularly otitis media (P2, P4, P18, and PX), and an inadequate vaccine response (PX, PX) were described, but, in CDdel cases, a complete analysis of the immune system has never been performed like in 22q11.2 DS [34].

## 6. Conclusions

The 3 Mb 22q11.2 “typical deletion” causes a fairly well-known clinical picture, while the effects of smaller CNVs harbored in this interval are yet to be fully elucidated.

In this review, we have collected all the information currently available on the deletion in the LCR22C-D interval, in order to obtain new insights for the classification of this CNV.

Thus far, data available on the genes, such as the HI score evaluation, HI predictors. and mechanisms of pathogenicity of genes associated with dominantly inherited disorders, are not exhaustive. The data on chromatin structure alterations due to this CNV are interesting but incomplete.

The review of the medical literature can give more clues about the role of this CNV. At first glance, CDdels are associated with a broad phenotypic spectrum, ranging from clinically normal to quite severe phenotypes; thus, predicting the clinical consequences of this CNV is still challenging. However, a thorough analysis of the frequency of specific clinical features highlights that renal/urinary tract abnormalities, cardiac defects, and neurological/behavioral disorders are much more common in CDdel than in the general population. This frequency is too high to be fortuitous, indicating that CDdel should not be considered a benign variant, but rather a predisposing factor for these phenotypic traits. The clinical management of CDdel cases include a careful evaluation of these aspects.

Among the genes present in this interval, CRKL is an excellent candidate for cardiac and renal defects, according to studies in humans and mice, whereas PI4KA could be a candidate for the neurological/behavioral disorder aspects. Even if further data are necessary to confirm the role of CDdels, according to our review, this CNV fits into the class of ‘likely pathogenic’ CNVs.

## Figures and Tables

**Table 1:** Genes included in LCR22C-D region.

Gene	Description	MORBID (Phenotype MIM Number)	HI Score	%HI	pLI
PI4KA *600286	phosphatidylinositol 4-kinase alpha	AR #619708 Gastrointestinal defects and immunodeficiency syndrome AR #616531 Polymicrogyria, perisylvian, with cerebellar hypoplasia and arthrogryposis AR #619621 Spastic paraplegia	N	35.17	0
SERPIND1 *142360	heparin cofactor II (serpin family D member1)	AD #612356 Thrombophilia 10 due to heparin cofactor II deficiency	N	68.88	0
SNAP29 *604202	synaptosomal-associated protein, 29 kd	AR # 609528 Cerebral dysgenesis, neuropathy, ichthyosis, and palmoplantar keratoderma syndrome (CEDNIK)	30	61.36	0.01
CRKL *602007	CRK like proto-oncogene, adaptor protein	N	1	4.35	0.45
AIFM3 *617298	apoptosis-inducing factor, mitochondrion-associated, 3	N	N	38.59	0
LZTR1 *600574	leucine zipper-like transcription regulator 1	AD # 616564 Noonan syndrome 10 AR #605275 Noonan syndrome 2 AD #615670{Schwannomatosis-2, susceptibility to}	N	32.82	0
THAP7 *609518	THAP domain- containing protein 7	N	N	50.87	0.01
P2RX6 *608077	purinergic receptor P2X-like 1	N	N	76.26	0
SLC7A4 *603752	solute carrier family 7, member 4	N	N	74.02	0
***LRRC74B*** N	leucine rich repeat containing 74B	N	N	N	0
***FAM246A*** N	family with sequence similarity 246 member A	N	N	N	N
RIMBP3B *612700	RIMS-binding protein 3B	N	N	89.37	0.41
HIC2 *607712	hypermethylated in cancer 2	N	N	59.73	1
***TMEM191C*** N	transmembrane protein 191C	N	N	N	N
RIMBP3C *612701	RIMS- binding protein 3C	N	N	88.68	N
UBE2L3 *603721	ubiquitin-conjugating enzyme E2 L3	N	N	3.65	0.87

For each gene, MIM number (*), phenotype MIM number (#), HI score evaluation, HI%, and pLI are reported. The phenotype associated with OMIM/MORBID genes (red) and the pattern of inheritance are showed. N = not available/reported, HI = haploinsufficiency score, HI% = DECIPHER haploinsufficiency index, pLI gnomAD v4.0 pLI score (https://www.ncbi.nlm.nih.gov/projects/dbvar/clingen/) (accessed on 28 November 2024) AR = autosomal recessive; AD = autosomal dominant.

**Table 2:** CDdel cases with pathological features.

Reference/ ID DECIPHER	Subjects	Sex/Age	Inheritance	Position (Mb)	Extent (kb)
Kurahashi et al., 1996 [21]	**P1**	NR/NR	NR	NR	NR
D’Angelo et al., 2007 [22]	**P2** P3: mother	F/8y F/NR	mat NR	NR	About 1000
Yu et al., 2012 [23]	**P4**: case11	M/NR	NR	20.725–21.205	480
	**P5**: case12	F/NR	NR	20.725–21.205	480
	**P6**: case13	M/NR	NR	20.725–21.205	480
Sanna-Cherchi et al., 2012 [24]	**P7**: ITA_25		inherited	NR	370-410
	**P8**: ITA-13		inherited	NR	370-410
	**P9**: Czec_1		dn	NR	370-410
Verhagen et al., 2012 [25]	(family A) **P10**: case 1	F/32y	NR	20.654–21.274	620
	(family C) **P11**: case 4 P12: case 5 (mother)	F/TP F/33y	mat NR	20.683–21.274	591
	(family D) **P13**: case 6	F/18y	dn	20.706–21.107	401
	(family E) **P14**: case 7 P15: case 8	M/5y F/37y	mat dn	20.706–21.107	401
Rump et al., 2014 [19]	(family 2) **P16**: case 4 P17: case 5 (mother)	M/7y F (47,XXX)/31y	mat NR	20.711–21.107	396
	(family 4) **P18**: case 7 P19: case 8 (father) P20: case 9 (brother) P21: case 10 (brother) P22: case 11 (grandfather)	M/8y M/38y M/5y M/13y M/68y	pat pat pat pat NR	20.699–21.151	452
	(family 8) **P23**: case16	M/12y	NR	20.656–21.574	918
	(family 10) **P24**: case18	M/4y	mat *	20.706–21.107	401
	(family 12) **P25**: case 21	M/11y	mat *	20.691–21.105	414
Racedo et al., 2015 [8]	**P26**	NR	NR	NR	NR
Williams et al., 2016 [26]	**P27**	F/6 w	NR	20.726–21.086	360
Lopez- Rivera et al., 2017 [9]	**P28**: patient 5	M/1m	NR	20.695–21.115	420
	**P29**: patient 6	M/TP	NR	20.705–21.115	410
	**P30**: patient 7	F/16y	NR	20.705–21.105	400
	**P31**: patient 8	F/9y	NR	20.705–21.105	400
	**P32**: patient 9	M/birth	NR	20.715–21.105	390
	**P33**: patient 10	M/birth	NR	20.725–21.115	380
	**P34**: patient 11	F/21y	NR	20.735–21.115	370
	**P35**: 12164-A	M/13m	NR	20.715–21.105	390
	**P36**: 12283-A	M/1y	NR	20.715–21.105	390
Clements et al., 2017 [27]	**P37, P38, P39, P40 (PX)**	3M-1F/ 2P <3y; 2P 15+y	NR	NR	NR
Gavril et al., 2022 [28]	**P41**	F/14y	dn	NR	NR
Stefekova et al., 2022 [29]	**P42**	NR/TP	NR	20.695–21.111	416
249397 (DECIPHER)	P43	F	inherited *	20.678–21.585	907
249400 (DECIPHER)	P44	F	NR	20.721–21.095	374
251146 (DECIPHER)	P45	M	NR	20.706–21.107	401
255749 (DECIPHER)	P46	M	inherited *	20.740–21.109	368
262738 (DECIPHER)	P47	F	NR	20.721–21.025	304
273516 (DECIPHER)	P48	M	inherited	20.400–21.086	686
279514 (DECIPHER)	P49	M	NR	20.713–21.111	397
504011 (DECIPHER)	P50	F	NR	20.727–21.086	359
519448 (DECIPHER)	P51	M	mat	20.740–21.109	368
424297 (DECIPHER)	P52	F	pat	20.721–21.100	379
501528 (DECIPHER)	P53	F	pat	20.722–21.103	380
501567 (DECIPHER)	P54	M	NR	20.722–21.103	380
339858 (DECIPHER)	P55	F	mat	20.721–21.109	388
390043 (DECIPHER)	P56	F	pat	20.721–21.109	388

For each case (P), age, sex and inheritance are reported along with the literature reference or the DECIPHER identification number (https://www.deciphergenomics.org) (accessed on 28 November 2024). In familiar cases, the proband is highlighted in bold. CDdel position (first and last abnormal probe) and extent are shown according to GRCh38/hg38. PX refers generically to one of the 4 individuals (P37, P38, P39, and P40) that is not possible to distinguish individually [26]. Two of these patients are under 3 years old and two are over 15 years old (2P < 3 y; 2P 15 + y) [26]. F: female; M: male; m: months; mat: maternal; pat: paternal; NR = not reported; asterisk (*) = inherited by asymptomatic parent; TP = terminated pregnancy.

**Table 3:** Macro-areas of pathological traits.

Macro-Areas	Frequency	Excluded in N Cases	Anomaly Description
**MORE FREQUENT**			
**Renal/urinary** **abnormalities**	**31.1%** (23/74) 65 + 9 = 74	N = 21	mono/bilateral renal agenesis (P16, P29, P33, P34, P35, PX, P42, P47, P54); renal hypodysplasia (P7, P8, P9, P28, P31, P32); hydronephrosis (P18, P19, P36, P52); vesicoureteral reflux (P30, P32, P33); renal cyst/s (P10, P22, P28); uretero-pelvic junction stenosis (P20, P32) pyelonephritis (P20); megaureter (P28); renal hyperchogenicity (P36).
**Cardiac defects**	**14.5%** (10/69) 65 + 4 = 69	N = 21	tetralogy of Fallot (P1, P10, P26, P36, PX); ventricular septal defect (P41, P51); atrio-ventricular septal defect (P17); pulmonary atresia (P1), pulmonary artery stenosis (P36), subpulmonary stenosis (P51), mitral valve incompetence (P17), dilated cardiomyopathy (P49), supraventricular tachycardia (P49), ventricular extra systole (P22).
**Neurological/behavioral disorders**	**52.1%** (37/71) 65 + 6 = 71		
DD/ID	**38%** (27/71) 27/65 + 6	N = 8	DD/ID (P4, P21, P54, P46, P48, nssv3466483, nssv3482339, nssv3472863, nssv3482015, nssv13648688, nssv580067); mild DD/ID (P10, P12, P17, P18, P25); severe DD/ID (P13, P14, P24); speech delay (P2, P5, P20, P35, P46); dyslexia (P18, P21, P23); learning disability (P2, P6, P19); motor delay (P15, P20, P25).
ASD	**4.2%** (3/71)		ASD(P25); PDD (P14); PDD-NOS (P21, P25).
ADHD spectrum disorder	**8.5%** (6/71)		ADHD (PX); hyperactivity (P2, P43, P44, P48); ADD (P18); short attention span (P43).
Behavioral and mood disorders	**7%** (5/71)	N = 9	psychosis (P43); aggressive and self-injurious behavior (P2, P18); hyperphagia, sleep problems (P2); OCD (PX); ODD (PX); depressive disorder (P3, PX); anxiety (P3, P18, PX).
Movement disorders	**9.9%** (7/71)		stereotypic movements (P13); chorea (P13, P25); limb ataxia (P13); clumsiness (P17, P18, P19, P21); tremor (P17, P18, P19, P22); cramps (P17).
“heterogeneous” neurological signs	**16.9%** (12/71)		hypotonia (P14, P18, P21, P25, P47); joint hypermobility (P13, P51, P56); hyperreflexia (P15, P17, P18); Babinski reflexes (P17); mild ptosis (P14, P15, P18); dysphagia (P36); sensorineural hearing impairment (P50).
**LESS FREQUENT**			
**Cleft palate/high arched palate**	**9.2%** (6/65)	N = 12	high arched palate (P2, P3); high narrow palate (P18, P19, P22); bilateral cleft lip and palate (P27).
**Genital anomalies**	**10.8%** (7/65)	N = 9	mono/bilateral cryptorchidism (P14, P16, P28, P33); phymosis (P20, P32); epididymis and ductus deferens agenesis (P16); uterus bicornis (P10).
**Skeletal anomalies**	-	N = 13	short stature (P12, P13, P23, P43, P45, P52); microcephaly (P5, P13, P27, P47); kyphosis (P17); scoliosis (P24); 6 lumbar vertebrae (PX); pes planus (P2); pes cavus (P17); 6 thoracic ribs (PX); missing canine (P23); hammered toes (P17); small hands (P2); craniosynostosis, ridged cranial sutures (P47); delay in bone (P23).
**Morbidity**	**-**		ear infections (P2, P18); recurrent infections (P14, PX, PX); inadequate vaccine response (PX, PX); low Ig (PX, PX).

The frequency of each trait is reported as the ratio between the number of affected cases and the total number of selected cases. A detailed description of each clinical sign and the number of individuals for whom the clinical trait has been actually evaluated and excluded is reported. PX refers generically to one of the 4 individuals (P37, P38, P39, and P40) that is not possible to distinguish individually [26]. ID: intellectual disability; DD: developmental delay; ADHD: attention deficit/hyperactivity disorder; OCD: obsessive compulsive disorder; ODD: oppositional defiant disorder; PDD: pervasive developmental disorder; PDD-NOS: pervasive developmental disorder not otherwise specified; ADD: attention deficit disorder; ASD: autism spectrum disorder.

## Data Availability

Data presented in this review are available since taken from published literature and public databases.

## Supplementary Materials

The following supporting information can be downloaded at: https://www.mdpi.com/article/10.3390/genes16010072/s1, Table S1: Detailed clinical features of 56 symptomatic individuals with CDdel; Table S2: Craniofacial dysmorphisms in CDdel cases; Table S3: Pathological signs that had been researched but not found in the cohort of 56 symptomatic individuals.
