# Supplementary material for: 22q11.21 Deletions: A Review on the Interval Mediated by Low-Copy Repeats C and D
Source: Genes (Basel). 2025 Jan 9;16(1):72. doi: 10.3390/genes16010072 (PMC11764475; doi:10.3390/genes16010072)
Supplement: Supplementary file 1 [file genes-16-00072-s001.zip › Table S1.pdf]

Cases	Phenotype features
P1	Tetralogy of Fallot, pulmonary atresia.
P2	Speech delay, learning disability, hyperactivity, aggressive and self-injurious behavior, hyperphagia, sleep problems, high-arched palate, small hands, pes planus, obesity, ear infections CFD. **Brain MRI:** abnormal signal in the posterior periventricular white matter.
P3	Depressive disorder and anxiety, high-arched palate CFD.
P4	DD.
P5	Speech delay, CFD.
P6	Mild learning disability CFD.
P7	Renal hypodysplasia.
P8	Renal hypodysplasia.
P9	Renal hypodysplasia.
P10	Renal cyst, uterus bicornis, tetralogy of fallot, mil DD, embryotoxon, CFD.
P11	IUGR, , unilateral talipes equinovarus, relatively narrow thorax, short limbs, stubby hands and feet, proximally placed thumbs, thin ribs, decrease vertebral ossification and thin long bones, CFD. **Brain MRI**: plexus choroıdeus cyst; intraventricular hemorrhage, supratentorial calcifications, abnormal neuronal migration and polymicrogyria .
P12	Mild DD, mild ID, short stature, bladder stones, myopia, CFD.
P13	Severe DD, severeID, stereotypic movements, chorea, limb ataxia, joint hypermobility, short stature, CFD SPECT (Singlephoton emission computed tomopraphy) scan: decreased basal ganglia perfusion.
P14	Bilateral cryptorchidism, severe DD, PDD, severe axial hypotonia, mild ptosis, recurrent infections, CFD. **Brain MRI**: small choroid plexus cyst, dilated perivascular spaces in the white matter and delayed myelination.
P15	Mild motor delay, hyperreflexia, mild ptosis.
P16	Unilateral renal agenesis, cryptorchid testis, epididymis and ductus deferens agenesis, CFD.
P17	Atrio-ventricular septum defect, mitral valve incompetence, mild DD, spastic paraplegia with an abnormal gait, clumsiness, tremor, cramps, increased tendon reflexes, Babinski reflexes, kyphosis, pes cavus, hammered toes, CFD.
P18	Mild left hydronephrosis, mild ID, ADD, anxiety disorder, aggressive behavioral outbursts, dyslexia, hypotonic face, ptosis of the left eye, clumsy motor skills, increased tendon reflexes, intention tremor, high narrow palate (nasal speech), recurrent ear infections, CFD.
P19	Mild hydronephrosis, learning disability, tremor, clumsy motor skills, CFD.
P20	Uretero-pelvic junction stenosis, pyelonephritis, phymosis, speech delay, motor delay.
P21	DD, (PDD-NOS), dyslexia, neonatal hypotonia, clumsy motor skills, CFD.
P22	Single renal cyst, ventricular extra systole, tremor, diaphragmatic hernia, high narrow palate, CFD.
P23	Dyslexia, delay in bone (SHOX normal) missing canine, single palmar creases, CFD short stature.
P24	Severe DD, scoliosis, CFD. **Brain MRI** spina bifida hydrocephaly, cerebellum agenesis .
P25	Motor delay, mild ID, (PDD-NOS) pervasive developmental disorder not otherwise specified, autism spectrum disorder (ASD), neonatal hypotonia, nonprogressive cerebral palsy, right-sided hemiplegia, dystonic and choreatic movements. **Brain MRI**: periventricular white matter changes. Prenatal or perinatal insult was suspected.
P26	Tetralogy of Fallot.
P27	Bilateral cleft lip and palate, CFD.
P28	Renal hypodysplasia, cortical cysts, left megaureter, monolateral cryptorchidism, chest hemangioma, CFD.
P29	Bilateral renal agenesis.
P30	Vesicoureteral reflux.
P31	Renal hypodysplasia.
P32	Renal hypodysplasia, uretero-pelvic junction stenosis, vesicoureteral reflux, bladder diverticuli, phymosis.

P33	Left renal agenesis, vesicoureteral reflux, monolateral cryptorchidism.
P34	Left renal agenesis.
P35	Right renal agenesis, speech delay.
P36	Renal hyperchogenicity, left hydronephrosis, tetralogy of Fallot, pulmonary artery stenosis, dysphagia.
P37-P40 (PX)	Monolateral renal agenesis (1/2); tetralogy of Fallot (1/2); ADHD (1/1), OCD (1/1), ODD (1/1), anxiety (1/1), MDD(1/1), low Ig, recurrent infection, inadequate vaccine response (2/4); 6 toracic ribs, 6 lumbar vertebrae (1/4).
P41	Ventricular setpal defects, growth delay, cafè au lait spots, hemangiomas, CFD.
P42	Bilateral renal agenesis.
P43	Primary amenorrhea, psychosis, short attention span, hyperactivity, aganglionic megacolon, proportionate short stature.
P44	Hyperactivity.
P45	Proportionate short stature, CFD.
P46	Delayed speech, ID, myopia, abnormal pinna morphology, abnormality of the middle ear ossicles, unilateral deafness, CFD.
P47	Renal agenesis, ID, hypotonia, CFD.<br>**Brain MRI**: hypoplasia of the corpus callosum.
P48	Hyperactivity.
P49	Dilated cardiomyopathy, supraventricular tachycardia.
P50	Sensorineural hearing impairment.
P51	Subpulmonary stenosis, ventricular septal defect, joint laxity.
P52	Hydronephrosis, CFD.
P53	Short stature.
P54	Renal agenesis, DD.
P55	Fetal cystic hygroma, hydrops fetalis.
P56	Joint hypermobility, CFD.

Table S1. Detailed clinical features of 56 symptomatic individuals with CDdel

PX refers generically to one of the 4 individuals (P37, P38, P39, P40) that is not possible to distinguish individually [26]. ID: intellectual disability; DD: developmental delay; CFD: craniofacial dysmorphisms; ADHD: attention deficit/hyperactivity disorder; OCD: obsessive compulsive disorder; ODD: oppositional defiant disorder; MDD: major depressive disorder; PDD: pervasive developmental disorder; PDD-NOS: pervasive developmental disorder not otherwise specified; ADD: attention deficit disorder; ASD: autism spectrum disorder; MRI: magnetic resonance imaging.
