# Supplementary material for: 22q11.21 Deletions: A Review on the Interval Mediated by Low-Copy Repeats C and D
Source: Genes (Basel). 2025 Jan 9;16(1):72. doi: 10.3390/genes16010072 (PMC11764475; doi:10.3390/genes16010072)
Supplement: Supplementary file 1 [file genes-16-00072-s001.zip › Table S2.pdf]

Cases	Craniofacial Dysmorphisms
P2	Narrow forehead, synophrys, upslanted palpebral fissures, deep-set eyes, small mouth and thin lips, short philtrum, retrognathia, micrognathia,high-arched palate strabismus.
P3	High-arched palate, similar facial features of P2 strabismus.
P5	Facial dysmorphisms not specified, microcephaly.
P6	Slightly rotated ears.
P10	High forehead, flat midface, long chin.
P11	Coarse facies, hypertelorism, small ears.
P12	Elongated face, prominent eyes, high nasal bridge with broad nasal tip, mild retrognathia.
P13	Microcephaly, wide mouth.
P14	Asymmetric facies, upward eye slant with short palpebral fissures, hypoplastic alae nasi, short philtrum, open mouth.
P16	Thin vermilion upper lip, overfolded helix left ear.
P17	Square face, upslanting palpebral fissures, overfolded helix, highy stature.
P18	High narrow palate (nasal speech), epicanthic folds, peri-orbital fullness, upturned nose, open mouth.
P19	Like son P18.
P20	NO facial dysmorphisms.
P21	Triangular face.
P22	Long face, short parpebral fissures, high narrow palate.
P23	Missing canine, triangular asymmetric face.
P24	Epicanthal folds, hypertelorism, macrocephaly due to ventriculomegaly.
P25	NO facial dysmorphisms.
P27	Bilateral cleft lip and palate, malar flattening, relatively small premaxillary segment microcephaly.
P28	Epicanthus.
P41	Facial dysmorphisms not specified.
P45	Downslanted palpebral fissures, facial asymmetry, round face.
P46	Prominent ear helix.
P47	Craniosynostosis, ridged cranial sutures, almond-shaped palpebral fissure, highly arched eyebrow, micrognathia, primary microcephaly.
P48	Abnormality of the face.
P51	Short nose.
P55	Short philtrum, synophrys, thin upper lip vermilion.

Tabella S2. Craniofacial dysmorphisms in CDdel cases
