# Supplementary material for: 22q11.21 Deletions: A Review on the Interval Mediated by Low-Copy Repeats C and D
Source: Genes (Basel). 2025 Jan 9;16(1):72. doi: 10.3390/genes16010072 (PMC11764475; doi:10.3390/genes16010072)
Supplement: Supplementary file 1 [file genes-16-00072-s001.zip › Table S3.pdf]

	**Cases for whom the abnormalities were excluded**	**Tot n° cases**
Renal/Urinary anomalies	P2, P12, P11, P13, P14, P15, P17, P21, P23, P25, PX, P41<br>(9 people reported by Lopez- Lopez-Riveira el al.,2017)	21
Heart anomalies	P2, P11, P12, P13, P14, P15, P16, P18, P19, P20,<br>P21, P23, P24, P25, P27, PX, P42<br>(4 cases reported by Racedo et al., 2015)	21
DD/ID	P3, P15, P16, P17, P22, P23, P27, P41	8
Behavioral disorder	P10, P12, P15, P16, P17, P19, P20, P22, P41	9
Cleft palate/high arched palate	P1, P10, P12, P13, P14, P15, P23, P24, P25, PX, PX, P41	12
Genital anomalies	P2, P11, P13, P15, P17, P21, P23, P25, P41	9
Scheletal anomalies	P2, P16, P18, P19, P20, P21, P22, P23, P25, P27, PX, PX, PX	13
Microcephaly	P16, P17, P18, P19, P20, P21, P22, P23, P24, P25, P41	11
Hypocalcemia	P1, P2, P10, P14, P15, P37, P38, P39, P40, P41	10
Immunodeficiency	P2, P41, PX, PX	4
Thymic hipoplasia	P1	1
Earing impairement	P2, P10, P12, P13, P14, P15, P37, P38, P39, P40, P41	11
Recurrent infections	P10, P12, P13, P15, P16 (ear), P17 (ear), P19 (ear), P20(ear),<br>P21(ear), P22(ear), P23(ear), P24(ear), PX, PX	14
Eye anomalies	P13, P14, P37, P38, P39, P40	6
EEG normal	P13, P14	2
Brain MRI normal	P13, P27	2
EMG (electromyogram normal)	P14, P17	2
Absent facial dysmorphisms	P20, P25	2

Tabella S3. Pathological signs that had been researched but not found in the cohort of 56 symptomatic individuals

For each clinical sign the total number of individuals actually evaluated is given. PX refers generically to one of the 4 individuals (P37, P38, P39, P40) that is not possible to distinguish individually [26].
